# Extended Preoperative Audiometry for Outcome Prediction and Risk Analysis in Patients Receiving Cochlear Implants

**DOI:** 10.3390/jcm12093262

**Published:** 2023-05-03

**Authors:** Jan-Henrik Rieck, Annika Beyer, Alexander Mewes, Amke Caliebe, Matthias Hey

**Affiliations:** 1 Medical Faculty, CAU Kiel, 24105 Kiel, Germany; 2 Audiology, ENT Clinic, UKSH Kiel, 24105 Kiel, Germany; annika.beyer@uksh.de (A.B.); alexander.mewes@uksh.de (A.M.); matthias.hey@uksh.de (M.H.); 3 Institute of Medical Informatics and Statistics, CAU Kiel, 24105 Kiel, Germany; caliebe@medinfo.uni-kiel.de

**Keywords:** cochlear implant, speech comprehension, prediction model, preoperative audiometric diagnostics, hearing loss, auditory rehabilitation, speech perception

## Abstract

Background: The outcome of cochlear implantation has improved over the last decades, but there are still patients with less benefit. Despite numerous studies examining the cochlear implant (CI) outcome, variations in speech comprehension with CI remains incompletely explained. The aim of this study was therefore to examine preoperative pure-tone audiogram and speech comprehension as well as aetiology, to investigate their relationship with postoperative speech comprehension in CI recipients. Methods: A retrospective study with 664 ears of 530 adult patients was conducted. Correlations between the target variable postoperative word comprehension with the preoperative speech and sound comprehension as well as aetiology were investigated. Significant correlations were inserted into multivariate models. Speech comprehension measured as word recognition score at 70 dB with CI was analyzed as (i) a continuous and (ii) a dichotomous variable. Results: All variables that tested preoperative hearing were significantly correlated with the dichotomous target; with the continuous target, all except word comprehension at 65 dB with hearing aid. The strongest correlation with postoperative speech comprehension was seen for monosyllabic words with hearing aid at 80 dB. The preoperative maximum word comprehension was reached or surpassed by 97.3% of CI patients. Meningitis and congenital diseases were strongly negatively associated with postoperative word comprehension. The multivariate model was able to explain 40% of postoperative variability. Conclusion: Speech comprehension with hearing aid at 80 dB can be used as a supplementary preoperative indicator of CI-aided speech comprehension and should be measured regularly in the clinical routine. Combining audiological and aetiological variables provides more insights into the variability of the CI outcome, allowing for better patient counselling.

## 1. Introduction

Cochlear implantation has made a significant impact on the treatment of profound hearing loss in recent decades [1,2]. While cochlear implants (CIs) were initially used for aiding profoundly deaf patients, the use of CIs has nowadays expanded to include the treatment of residual hearing [3,4,5], asymmetric hearing loss (AHL) [6] and single-sided deafness (SSD) [7]. Until a short while ago, the audiological indication for receiving a CI was ≤30% monosyllabic comprehension at 70 dB sound pressure level (dB_SPL_) with optimum hearing-aids. However, in recent years the range of indications has expanded considerably [8,9,10]. Today, for postlingually deaf adults, a preoperative speech comprehension of ≤60% at 65 dB_SPL_ with optimally adjusted conventional hearing aids is considered one of the main indications for CI implantation [8,9,10]. This has resulted from improvements of CI electrodes, surgical technology and speech-processor technology [10,11,12,13]. Implantation is now a standard procedure [10,12] and is considered safe in terms of complication rates [14,15]. Nonetheless, the implantation of a CI remains an invasive procedure with the possibility of complications [1,14,16].

It has been demonstrated that most patients show improved hearing with a CI. In a study conducted by Hoppe et al. [17], 98.4% of patients who received a cochlear implant showed an improvement in their ability to comprehend monosyllabic words at 65 dB_SPL_ compared with their performance with hearing aids before implantation. Postoperatively, 97% of patients achieved at least their preoperative maximum word recognition score (WRSmax; WRS is expressed in %) [17]. However, since in some cases little or no improvement in speech comprehension is observed, it is desirable to have valid criteria to weigh the risk-benefit profile in individual patients and to estimate the potential postoperative outcome before implantation.

In clinical practice, preoperative diagnostics and anamnesis are used as a predictive basis for the postoperative result. In German-speaking countries, the Freiburg monosyllabic word test and the two-digit number test are used as audiological criterion for indication of CIs and for postoperative evaluation of performance [18,19]. The Freiburg monosyllabic test is a standardised, easy-to-use and critically reviewed test procedure [18,20,21,22]. For that test, Hoppe et al. have shown that unaided maximum word recognition (WRSmax) can serve as a predictor for the minimum expected benefit with a CI. Furthermore the monosyllabic speech comprehension at 65 dB_SPL_ with a hearing aid (WRS65(HA)) can be used as well [4].

In 2012, Blamey et al. published criteria independent of speech audiometry for postoperative speech comprehension [23]. In their multicentre cohort, a positive correlation was found between the time elapsed since CI implantation and postoperative speech comprehension. Regarding the latter, the duration of hearing loss, age at cochlear implant (CI) fitting, age at onset of hearing loss, as well as certain conditions such as meningitis, temporal bone fracture, and auditory neuropathy spectrum disorder (ANSD) were found to be negatively correlated with the postoperative speech comprehension. On the other hand, the outcome was positively correlated with genetic aetiology and the presence of Ménière’s disease. Combining the duration of severe to profound hearing loss, age at onset of hearing loss, aetiology and duration of CI experience, Blamey et al. were able to explain 10% of the postoperative variation in their model [23]. An updated model with more influencing factors, including the pure-tone average, raised the explanatory power for postoperative speech comprehension to 22% within the same patient collective [24]. Furthermore, a large multicentre study by Goudey et al. [25] with 2735 patients revealed a variation of 12–21% for factors influencing the postoperative outcome, including speech comprehension measures. The variation between the cohorts at each study location was noted. It was found that, as expected, a smaller study cohort and fewer criteria in the multivariate analysis resulted in a poorer explanation of the postoperative variation [25].

In a monocentric study by Kraaijenga et al. [26] with 88 prelingually and 370 postlingually CI-implanted patients, a postoperative variation of 26% was accounted for. The model showed a positive correlation between the postoperative outcome and (i) preoperative speech comprehension with optimum hearing-aid fitting and (ii) age at onset of deafness. A negative correlation was found between speech comprehension and meningitis [26]. These results demonstrate that it appears reasonable to include etiological aspects, on the basis of their correlation with speech comprehension. It should be noted that the positive correlation of age at onset of deafness with speech comprehension, found by Kraaijenga et al., contrasts with the above-mentioned finding of Blamey et al. [23]. In a study by Hoppe et al. [17] the opposite behaviour was shown too. With regard to postoperative speech comprehension at 65 dB_SPL_ CI, a logarithmic model was used to include not only WRSmax and WRS65(HA), but also age at implantation. The median absolute error indicating the average of the absolute difference between speech comprehension as predicted and as measured postoperatively was 13.5 percentage points [17]. Green et al. [27] were able to explain a postoperative variation of 9% with a much smaller cohort (117 patients). Against this background, more clarification of the postoperative variation is needed [2].

In the present study we examined preoperative pure-tone audiogram and speech comprehension as well as aetiology, in order to investigate their relationship with postoperative speech comprehension in CI recipients. Specifically, the aims of this work were:

- To assess whether including preoperative word comprehension at 80 dB_SPL_ with a hearing aid improved the prediction of postoperative speech comprehension with a CI. This was also considered in relation to preoperative maximum word recognition.
- To investigate whether preoperative two-digit number comprehension and the four-frequency pure-tone average of the frequencies 0.5, 1, 2, 4 kHz (4FPTA) are relevant factors for postoperative outcome with CI.
- To set up a multivariate model based on an extended dataset of preoperative audiometric diagnostics and thus to predict postoperative speech comprehension with a CI.

## 2. Materials and Methods

### 2.1. Patients

In this monocentric, retrospective study, data for 664 ears of 530 adult CI recipients were investigated after anonymisation. The inclusion period for the CI implantations was 2002–2019 inclusive. Exclusion criteria were CI surgery before the age of 18 years and displacing or malignant tumour in the area of the auditory pathway. Patients in this study underwent cochlear implantation with a CI24RE(CA), CI512, CI532, CI612 or CI632 implant type (Cochlear Ltd., Sydney, Australia). The speech processors used were models CP810, CP910 and CP1000, depending on the date of implantation and technical availability. A positive vote by the Ethics Committee at the Faculty of Medicine of Kiel University (study number D 455/20) was obtained.

The collection of anonymised anamnestic data at Kiel University Hospital was based on paper and digital files, as well as on the audiometry measurement system “evidENT” (Merz Medizintechnik, Reutlingen, Germany).

On the basis of each patient’s medical diagnosis and history of hearing loss, 13 aetiological categories were created as follows:

- Unknown: no specific disease
- Middle ear: cholesteatoma, surgery of the stapes, otitis media, otosclerosis
- Congenital: unspecified congenital hearing loss and hypoxia at birth
- Trauma: external forces, such as accident, acoustic trauma or occupationally related repeated acute exposure to damaging sound levels
- MMR: mumps, measles, rubella
- Genetics: family connection and/or medical diagnosis or suspicion of relevant hereditary factors
- General infection: post-infection condition and temporal relationship with the onset of hearing loss
- Syndromic complexes: syndromic disorders, (e.g., Mondini, Wolfram or Cogan syndrome)
- Ototoxic treatments: chemotherapy, or substances such as gentamycin.
- Idiopathic sudden sensorineural hearing loss identified in medical and audiological diagnosis
- Meningitis
- Ménière’s disease
- Miscellaneous: rare individual cases including enlarged vestibular aqueduct (EVA) syndrome, cerebral haemorrhage and microcephaly.

### 2.2. Audiometry

All patients had been assessed before and after CI implantation according to the standard procedures at our institution. In brief: To compare post- with preoperative hearing performance, hearing threshold was determined by pure-tone audiometry, and speech comprehension was measured by the Freiburg monosyllabic and two-digit number tests. The monosyllabic word test employed to measure speech comprehension [19,28] comprises 20 groups of 20 nouns each; the number test comprises multisyllabic two-digit numbers in 10 groups of 10 numbers each (e.g., 32 was read as “zweiunddreißig”).

In addition to these preoperative measurements with air-conduction headphones, speech comprehension in free field with hearing aids was also assessed.

The preoperative data that were extracted from the records comprised of several tests. These included:

(i) the pure-tone audiometry under air conduction at 500, 1000, 2000, and 4000 Hz,
(ii) the Freiburg monosyllabic test under air conduction without a hearing aid at speech levels 65, 80, 95, 110, and 120 dB_SPL_,
(iii) the Freiburg monosyllabic test in free field with a hearing aid at 65 and 80 dB_SPL_. For the Freiburg two-digit numbers, the sound levels were adjusted individually in the range 30–120 dB_SPL_ in 5 dB_SPL_ steps.

All audiometric measurements were performed monaurally with the ear to receive the implant, while the contralateral ear was masked when necessary. Speech comprehension in free field was performed monaurally, under best aided conditions, with the ear to receive the implant; the contralateral ear was masked when necessary. The 4FPTA was calculated from the pure-tone audiometry data as the mean value of the hearing threshold at 500, 1000, 2000 and 4000 Hz. In addition, the Freiburg monosyllabic test was used to determine the maximum monosyllabic word recognition WRSmax and the maximum two-digit number recognition NRSmax (both in %) as well as the speech reception threshold (SRT) for the two-digit number test. WRSmax and the NRSmax each represented the maximum measured ‘words correct’ and maximum measured ‘numbers correct’ score, both expressed as percentages. The SRT is defined as the minimum level at which 50% of the speech material is intelligible.

Postoperative, speech comprehension for the Freiburg monosyllabic words and for the two-digit numbers was recorded 2 years after first activation of the CI. In cases where no data were available for that time point, the 1.5-year values were used. The two-year mark was chosen, because it may be taken to provide a more reliable and accurate measure of the long-term success of the treatment, considering individual variability and environmental influences, even though stable outcomes may be expected one year after implantation. For the Freiburg monosyllabic words, speech comprehension was measured at speech levels between 40 and 80 dB_SPL_ in 10 dB_SPL_ steps, and for the two-digit number comprehension at speech levels between 25 and 85 dB_SPL_ in 5 dB_SPL_ steps. Concerning the words, the value for speech comprehension at 70 dB_SPL_ was used for further analysis.

### 2.3. Statistical Methods and Data Analysis

#### Univariable Analyses

Univariable analyses were performed and figures created by using IBM^®^ SPSS Statistics (IBM, Armonk, New York, NY, USA). R software version 4.0.3 was used for the regression models [29]. All tests were performed two-sided, and a significance level of 0.05 was chosen. The primary outcome of this study was the Word recognition score at 70 dB_SPL_ with CI (WRS70(CI)).WRS70(CI) and all continuous influence variables showed significant differences from a normal distribution (Shapiro–Wilk test, *p* < 0.001). Continuous variables were described by median and interquartile-range as well as mean in the descriptive analysis while for categorical variables absolute frequencies and percentages are given.

The outcome WRS70(CI) was investigated in two different ways. On the one hand, the continuous (but not normally distributed) measured values were used. On the other hand, WRS70(CI) was dichotomised (values below 1st quartile vs values above 3rd quartile). The first approach has the advantage of incorporating all the data and using all information from the measured values but cannot be analysed by a standard multiple linear regression model due to the lack of a normal distribution. The second one focuses on well-separated groups of ‘good’ and ‘poor’ performers and can be used by the established logistic regression model, while having to drop half of the data (between 1st and 3rd quartile). Because each of the approaches have unique advantages, we chose both of them for our statistical analyses.

For the comparison of WRS70(CI) between the different aetiological categories, Wilcoxon rank sum test was applied for continuous WRS70(CI) and the χ^2^ test for dichotomous WRS70(CI). The correlation between the continuous WRS70(CI) and preoperative four-frequency pure-tone average (4FPTA), maximum word recognition score (WRSmax), comprehension at 65 dBSPL with hearing aid (WRS65(HA)) and comprehension at 80 dBSPL with hearing aid (WRS80(HA)) was calculated using the Spearman correlation coefficient together with the corresponding test of the null hypothesis of no correlation. For dichotomous WRS70(CI) differences between good and poor performers in 4FPTA, WRSmax, WRS65(HA) and WRS80(HA) were tested with the Wilcoxon rank sum test. The same analysis strategy a was applied when analysis the influence of preoperative maximum two-digit number recognition score (NRSmax), and preoperative SRT of two-digit numbers without a hearing aid on WRS70(CI).

To adjust the significance level for multiple testing, the Bonferroni correction was applied.

### 2.4. Multiple Regression Models

Corresponding to the two scalings of our outcome WRS70(CI) (continuous but not normally distributed and dichotomous) we applied two different regression methods.

For dichotomous WRS70(CI) we applied multiple logistic regression. For continuous WRS70(CI), a standard multiple linear model could not be applied because of the lack of normality. The second approach utilised the percentage values of WRS70(CI) as outcome in a quasibinomial model. The quasibinomial model is especially suited for outcomes measured as percentage values (or proportions). The outcome is then modelled as the result of multiple binomial trials. 

Influence variables that showed a significant effect in the univariable analyses of WRS70(CI) were used as independent variables for both approaches. The variables included in the logistic regression analysis were WRS65(HA), WRS80(HA), WRSmax, 4FPTA, NRSmax, and the 50% number threshold, as well as the etiology variables meningitis, congenital (and hypoxic) etiology, and sudden idiopathic hearing loss. In addition, the age of the user at the time of hearing aid fitting and the duration of wearing a hearing aid or cochlear implant were included as variables. Not all the variables were included in the final model; only those that showed a significant association with postoperative outcome were selected. These variables had already been identified before the logistic regression. The age and duration variables were quantified by assigning to each CI recipient a number based on the quartile in which their respective scores fell (e.g., 1 for the lowest quartile and 4 for the highest quartile). Model selection was performed with backward-selection according to the likelihood ratio test. The models with and without the influence variable in question were compared with the ‘anova’ command and the influence variables with the highest *p* value were removed iteratively up to a *p* value threshold of 0.05. For evaluating the model performance, R^2^ values were calculated with the R package rsq [30]. For logistic regression, the standard R^2^ of linear models cannot be calculated. Nagelkerke’s R^2^ is therefore the established alternative for generalized linear models such as logistic regression. For quasi-models, the situation is more complicated because neither the standard R^2^ for linear models nor Nagelkerke’s R^2^ for generalized models can be applied. We therefore used the Kullback–Leibler divergence-based R^2^ as an alternative for measuring the model performance. The higher this R^2^, the better is the model fit. The Kullback–Leibler divergence-based R^2^ is a generalisation of McFadden’s R^2^ [31]. It is extended to quasi-models via the quasi-likelihood function [32].

## 3. Results

**Figure 1:**
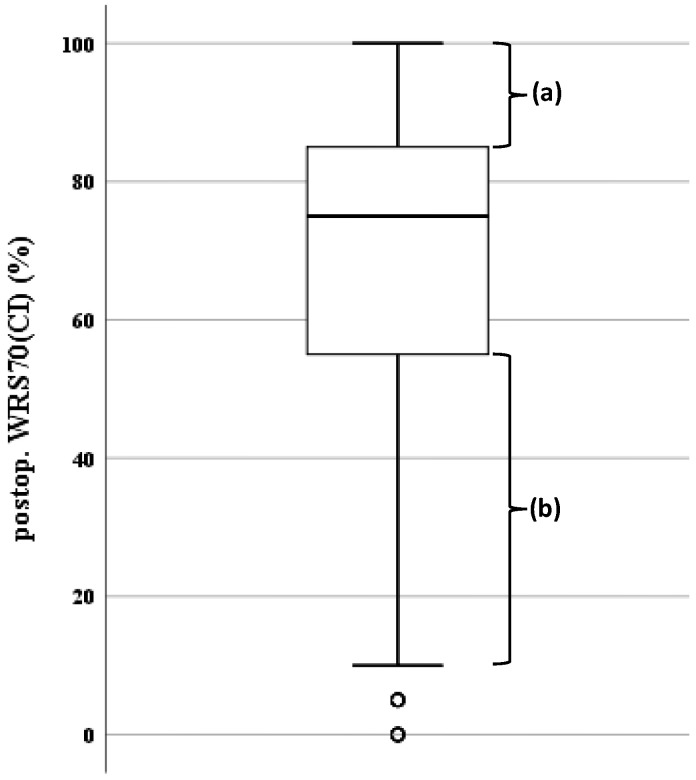
Boxplot of postoperative WRS70(CI). Representation of the median, the first and third quartiles, the minimum, maximum and outliers; the lower whisker extends to 1.5 times the interquartile range (IQR) and the upper whisker to the 100% limit. The bracket (a) indicates WRS70(CI) values above the 75th percentile (‘good performers’) and the bracket (b) values below the 25th percentile (‘poor performers’).

Preoperatively acquired data were compared with the postoperative monosyllabic word score at 70 dB_SPL_ measured with the CI activated (WRS70(CI)); the latter is considered one of the key performance indicators in the audiology department of the ear, nose and throat clinic of the UKSH Kiel for the clinical CI-fitting process [33]. This target variable was viewed in two ways: (i) as a continuous variable and (ii) as a categorical (dichotomous) variable. The aim of the second was to focus upon higher and lower performance level in speech comprehension. The boxplot in Figure 1. shows the dichotomisation of WRS70(CI). The 1st quartile corresponded to a WRS70(CI) of 55% and the 3rd quartile to a WRS70(CI) of 85%. The patients below the 1st quartile (‘b’ in the diagram) were termed ‘poor performers’ and the patients above the 3rd quartile (‘a’) ‘good performers’. Table 1 lists further characteristics of the patients’ audiometric data.

### 3.1. Aetiology

**Figure 2:**
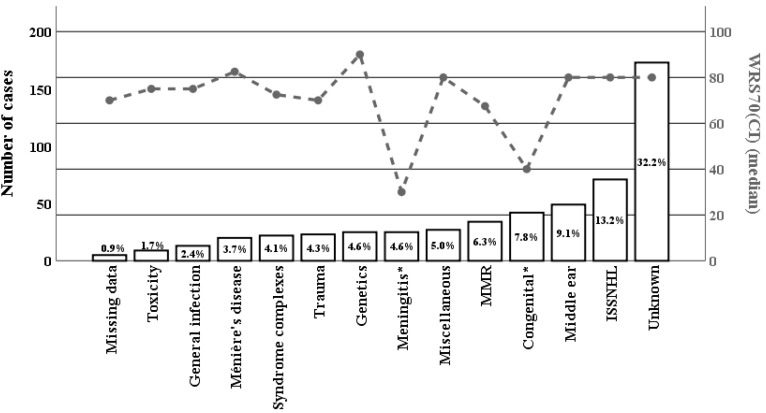
Absolute numbers and percentages of patients by aetiology of hearing loss in the ear that received the CI (left ordinate). The associated WRS70(CI) values in absolute numbers are also shown (right ordinate). For each aetiology the postoperative hearing assessment (median WRS70(CI)) is shown, as indicated by the dashed line. MMR, mumps-measles-rubella. Miscellaneous, data that could not be ascribed to a single category. ISSNHL, Idiopathic sudden sensorineural hearing loss. * Significant correlation (*p* < 0.01) in the Wilcoxon rank sum test after Bonferroni correction for multiple testing.

Figure 2 shows the distribution among the aetiological categories. The numerically largest category was ‘unknown’, at 32.2%. This was followed by ‘idiopathic sudden sensorineural hearing loss’ (ISSNHL, 13.2%), ‘middle ear’ (9.1%) and ‘congenital’ (7.8%). Although the category ‘unknown’ was large, it was in fact smaller than frequently reported (typically 44–62% [23,34,35]). In addition, the medians of WRS70(CI) (postoperative speech comprehension) are plotted for each category. Relatively low medians of WRS70(CI) are seen for ‘meningitis’ and ‘congenital’, with relatively high medians for ‘genetics’ and ‘Ménière’s disease’.

For the continuous WRS70(CI), Wilcoxon’s rank test was used to determine whether any aetiological category had a significant influence on the outcome of the CI implantation. Significant associations were found for congenital hearing loss (*p* < 0.001) and meningitis (*p* < 0.001) (after adjustment for multiple testing).

**Figure 3:**
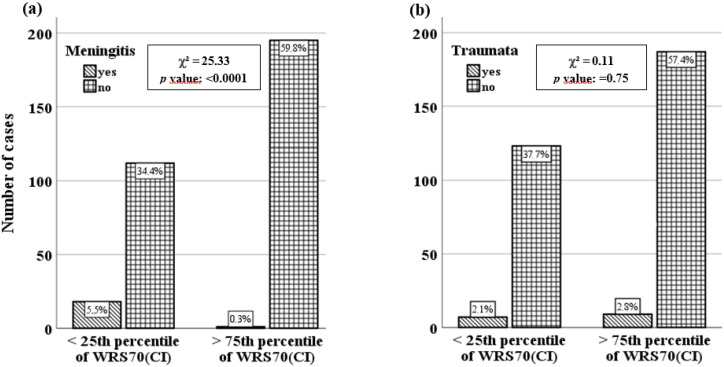
Two examples of the distribution of aetiology (present ‘yes’ or ‘no’) in relation to the dichotomous outcome measure above the 75th percentile (good performer) and below the 25th percentile (poor performer). (**a**) ‘Meningitis’, showing significant association with the dichotomous WRS70(CI); (**b**) ‘trauma’, showing no significant association. Statistics from the χ^2^ test are shown.

For the dichotomous characterisation of WRS70(CI), the test was applied. Figure 3 shows ‘meningitis’ and ‘trauma’ as examples. Among the aetiological categories reviewed, ‘meningitis’ (*p* < 0.001) showed the most clearly significant association with good/poor performer status. In contrast, there was no significant association between the aetiology ‘trauma’ and good/poor performer status (*p =* 0.75). In addition to ‘meningitis’, a significant association was found for ‘congenital hearing loss’ (*p* < 0.001) and ‘ISSNHL’ (*p* < 0.003) (Table 2) after adjustment for multiple testing by using the Bonferroni correction (*p*-value: 0.05, number of categories: 14, 0.05/14 = 0.0035 (Bonferroni-corrected alpha error)).

### 3.2. Preoperative Speech Comprehension

**Figure 4:**
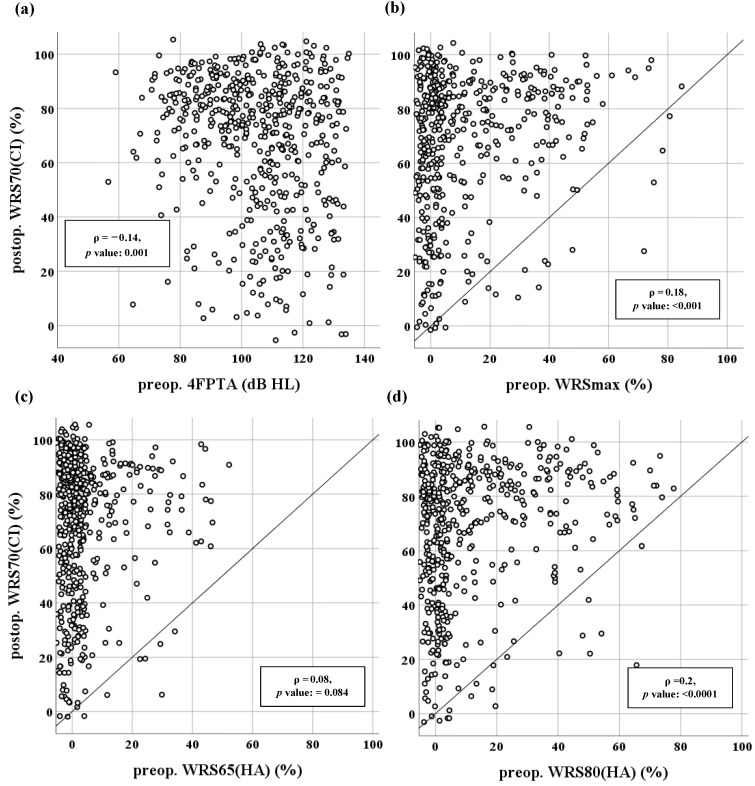
Postoperative speech comprehension with CI at 70 dB_SPL_ (WRS70(CI)) as a function of preoperative (**a**) four-frequency pure-tone average (4FPTA), (**b**) maximum word recognition score (WRSmax), (**c**) comprehension at 65 dB_SPL_ with hearing aid (WRS65(HA)) and (**d**) comprehension at 80 dB_SPL_ with hearing aid (WRS80(HA)). Points above the reference line (y = x) indicate better speech comprehension with the CI in comparison with the preoperative state. ρ: Spearman’s rank correlation coefficient. (**a**) *n* = 534, (**b**) *n* = 484, (**c**) *n* = 516, (**d**) *n* = 512. Data are presented as jitter plots (see text).

For the continuous analysis, WRS70(CI) is displayed in relation to preoperative 4FPTA, WRSmax, WRS65(HA) and WRS80(HA) in scatter plots (Figure 4). In Figure 4b–d, a reference line is inserted for speech comprehension to indicate whether a patient understood better or worse with the CI than preoperatively; values above this line signify an improvement in comprehension due to the CI. The smaller scatter width in the representation of the WRS65(HA) (Figure 4c) in contrast to the WRSmax and WRS80(HA) (Figure 4b,d) is striking. Accordingly, WRSmax and WRS80(HA) showed a greater proportion with a preoperative comprehension of greater than zero percent.

The respective Spearman rank correlation coefficients (ρ) are given in Figure 4. The estimated correlations are relatively low with the strongest correlation between WRS70(CI) and WRS80(HA) of around 0.2. 

In the dichotomous analysis, a significant difference in WRS65(HA) was found between the two groups of good and poor performers (Wilcoxon rank sum test: *p* < 0.01). Furthermore, 4FPTA, WRSmax and WRS80(HA) also showed a significant difference between good and poor performer groups, in addition to their association with WRS70(CI) in the continuous analysis (Wilcoxon rank sum test: *p* < 0.001).

Table 3 lists the results of the comparison between the measures of preoperative speech comprehension (WRSmax, WRS65(HA), WRS80(HA)) and the postoperative continuous WRS70(CI). The data show in how many cases (absolute and %) a postoperative improvement measured for WRS70(CI) could be achieved.

The cases in Table 3 for which WRS70(CI) was lower than the respective preoperative measure of speech comprehension (13, 5 and 11 ears respectively) were reviewed individually with respect to two points. First, the test–retest reliability of the monosyllabic test was to be taken into account. On the basis of the findings of Winkler and Holube [36] for the 95% confidence interval regarding the deviations of speech comprehension from the “true” value for 20 words per test list, we found that for WSRmax(%) 7 of 13 ears, for WRS65(HA) 1 of 4 ears and for WRS80(HA) 5 of 12 ears demonstrated poorer speech comprehension after CI fitting (poorer = outside the 95% confidence interval). This reduced the number of cases with demonstrated decreased speech comprehension in the WRS70(CI), as some were within the 95% confidence interval of the test–retest reliability. The second aspect was the patients’ fluctuating daily performance. WRS70(CI) was measured at earlier and later time points, if the first data review still showed a worse speech comprehension after CI-fitting and the actual 2-year value deviated from the average performance of the patient. Results arising from consideration of both these factors are shown Table 3 (last column). We found the following numbers of cases for the absolute percentage improvement between preoperative WRS80(HA) and postoperative WRS70(CI): 10%, 482; 20%, 454; 30%, 430; 40%, 373; 50%, 337; 60%, 267; 70%, 204; 80%, 128; 90%, 59; and 100%, 18.

**Figure 5:**
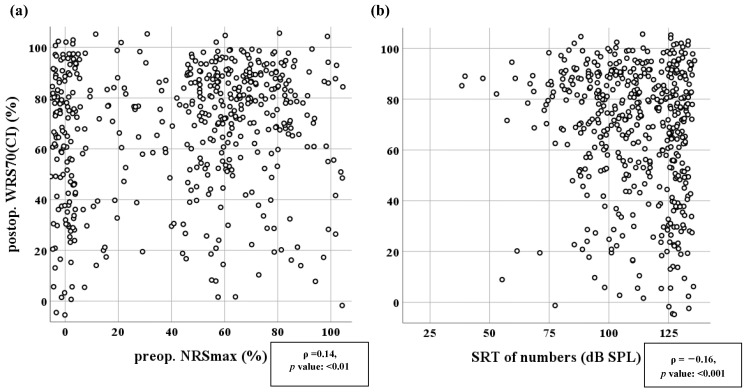
Postoperative speech comprehension with CI at 70 dB_SPL_ (WRS70CI) depending on (**a**) preoperative maximum two-digit number recognition score (NRSmax), and (**b**) preoperative SRT of two-digit numbers without a hearing aid. Statistics of the Spearman rank test are given. (**a**) *n* = 493, (**b**) *n* = 492. Data are presented as jitter plots (see text).

The postoperative WRS70(CI) was analyzed by comparison with preoperative speech comprehension for German two-digit numbers, assessed without a hearing aid. For this purpose, scatter plots were created (Figure 5) showing this relationship for preoperative maximum two-digit number comprehension and for preoperative SRT for such numbers. Considering the WRS70(CI) as the target value, the preoperative NRSmax and SRT of numbers in Figure 5 showed a wide spread of data. The significant results shown in Figure 5, obtained by using the continuous form of the WRS70(CI), were also confirmed by the CI recipients’ good/poor performer status according to the categorized WRS70(CI). Both the NRSmax and the SRT of numbers were significantly associated with the categorized WRS70(CI) (Wilcoxon rank sum test, *p* < 0.001). It is important to note that Figure 4 and Figure 5 are shown as jitter plots so that points do not overlap; consequently, as can be seen in Table 1, values are often at or below zero percent.

### 3.3. Merging the Predictors—Prediction via Multiple-Regression Models

Two regression models are presented. Both were based on the variables that showed significance in the univariable tests. The outcome variable in both models was WRS70(CI). This outcome was considered on two different scales: (i) The binary logistic regression analysis uses the highest and lowest quartile of the WRS70(CI). (ii) In contrast, by using a quasibinomial model, the precise WRS70(CI) percentages on the whole dataset can be handled.

The final binary logistic model using a maximum-likelihood backward selection is shown in Table 4. The highest quartile includes 198 ears and the lowest 131 ears. The odds ratios (OR) were related to a negative outcome. Nagelkerke’s R² was 0.40. Categories with OR < 1 were to be interpreted as indicators of reduced risk of a poor outcome, while OR > 1 implies an increased risk of a poor outcome. Risk factors for a poor outcome were found to be meningitis and congenital hearing impairment/hypoxia. Meningitis increased the risk of a poor outcome approximately 50-fold. On the other hand, the indicators of reduced risk were ISSNHL, a higher quartile in terms of age at first fitting of a hearing aid (compared with the 1st quartile as a reference), increasing WRS80(HA) and NRSmax. As an example, for each percentage-point increase in WRS80(HA), there was a reduction by a factor of 0.966 in the risk of a poor outcome. For comparison with the quasibinomial model, the Kullback–Leibler divergence-based R² was calculated. The value was 0.26.

The quasibinomial model is shown in Table 5. 538 ears were included on the basis of WRS70(CI). It was not possible to calculate Nagelkerke’s R² in this case, so here too the Kullback–Leibler divergence-based R² was calculated. It was 0.23. The regression coefficients referred to a high WRS70(CI). Consequently, values with a positive sign were predictive of good postoperative comprehension. An increasing WRS65(HA), meningitis disease and congenital hearing impairment/hypoxia had a negative association with WRS70(CI). In contrast, an increasing WRS80(HA) and a higher quartile in terms of age at first fitting of a hearing aid were positively associated with WRS70(CI). Regarding the duration of wearing a hearing aid, the patients above the 3rd quartile were taken as a reference. Here, patients in the 2nd–3rd quartile showed a significantly higher WRS70(CI) percentage than patients above the 3rd quartile. Consequently, shorter wearing time (2nd–3rd quartile) was positively associated with CI outome compared to reference (above 3rd quartile).

**Figure 6:**
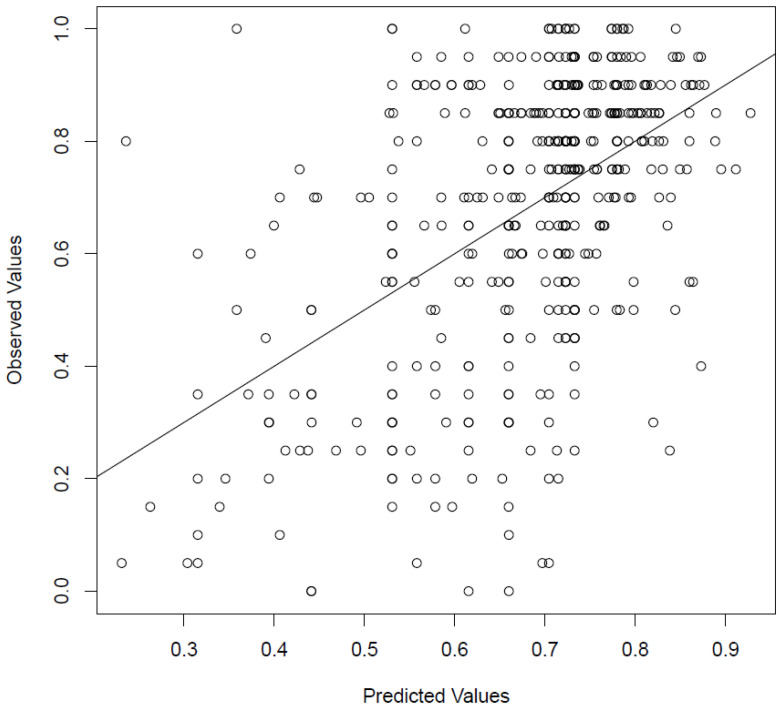
Comparison between observed and predicted values of WRS70(CI) based on the quasibinomial model (*n* = 538). Decimal values correspond to percentages (e.g., 0.3 = 30%). The line corresponds to equality between measured values and the model. Accordingly, values above this line were underestimated in the prediction and values below it were overestimated.

On the basis of the quasibinomial model, the median absolute error for the prediction of WRS70CI could be calculated. It was 13.8%. This is illustrated graphically in Figure 6. For this purpose, the collected WRS70(CI) measurements were plotted against the expected values based on the quasibinomial model.

## 4. Discussion

### 4.1. Speech Comprehension

Nowadays, most adult patients who receive a cochlear implant experience a significant improvement in speech comprehension after surgery [4,26,37]. This is consistent with the results of the present study. Here, 97.3% of CI-provided ears performed better than or as well as preoperative performance measured by WRSmax. This was the case in as many as 99.2% of ears when WRS65(HA) was the preoperative criterion. This is comparable to the results of Hoppe et al. who reported that the postoperative variable WRS65(CI) was greater than or at least equal to the preoperative WRSmax in 96% of CI recipients [4]. In another study published by Hoppe et al. [17] it was found that under the condition that preoperative speech comprehension was above 0%, 98.4% of these patients had a WRS65(CI) score that was either the same as or higher than their preoperative WRS65(HA) score.

In our study, WRS80(HA) was also considered. With the CI, 97.7% of the ears in the postoperative measurement at 70 dB_SPL_ achieved or surpassed the preoperative WRS80(HA). In addition to WRSmax, used in studies by Hoppe et al. [4], WRS80(HA) could be used as a predictor of minimum postoperative speech comprehension. Compared with WRS65(HA), the use of WRS80(HA) is justified by a lower percentage of ears with no preoperative speech comprehension (0%) and a higher overall mean value of preoperative speech comprehension (Table 1, Figure 4). Preoperative WRSmax and WRS80(HA) showed comparable mean values and also approximately similar values in terms of numbers of ears with 0% speech comprehension. However, the correlation of WRS80(HA) with WRS70(CI) proved to be stronger than that of WRSmax with WRS70CI. Reference data regarding the latter variable are, to the best of our knowledge, not available in the literature.

WRSmax is discussed by Halpin et al. [36] as a possible indicator of cochlear damage as well as of impaired capacity to carry the information that is responsible for word recognition [4,38]. Furthermore, those authors mention that hearing aids may be beneficial if residual speech comprehension is present, up to the limit imposed by the cochlear damage. This means that hearing aids have the potential to improve speech comprehension to some degree, depending on the severity of the cochlear damage. Thus, the fact that the correlation between WRS70(CI) and WRS80(HA) was found to be higher than that between WRS70(CI) and WRSmax may have been due to a technical adjustment of the auditory impression by the hearing aid [36,38,39]. Technical adjustments can include modifications to the sound wave picked up by the device, such as volume adjustments, tone adjustments and suppression of disturbing noises, to enhance the listening experience for the hearing aid wearer. The weaker correlation between WRS65(HA) and WRS70(CI) may have been caused by the hearing aid’s amplification mechanism [39], resulting in insufficient amplification at 65 dB_SPL_ input volume when a high degree of hearing loss was present. In this context, inadequate CI fitting (resulting in the need for higher sound pressure levels for word comprehension) or possible technical difficulties should be considered [40,41,42].

The Freiburg two-digit number test uses NRSmax and SRT of numbers. The idea behind its introduction was to include a speech-audiometric test procedure that would reveal fewer cases where no residual comprehension was measurable under air conduction. Consistently with this, the present study showed that there were fewer cases with no speech comprehension when the two-digit number test was used than when the monosyllabic test was used (Table 1). For the NRSmax (ρ = 0.14) and the SRT (ρ = 0.16) the correlations with the postoperative outcome WRS70(CI) were weaker than for WRS80(HA) and WRSmax. While the final binary logistic model explained part of the postoperative variation using NRSmax, it should be noted that two-digit number comprehension is not a sufficient indicator of the overall benefit of cochlear implantation in daily life, as pointed out by Müller-Deile [43], because 100% two-digit number comprehension can often be expected at the initial CI fitting. However, with each new piece of relevant information, it is possible to develop a more comprehensive model that explains a significant portion of the variability in postoperative speech comprehension.

It should be noted that about 2.7% of ears did not show an improvement in WRSmax compared with comprehension using a CI (Table 3). This proportion was lower after taking into account the test–retest reliability of the Freiburg monosyllabic test and the patient’s day-to-day performance fluctuation. Our value was in agreement with the literature, where a range of 3–4% has been reported [4,23,44]. These poorly performing ears exemplify the need for improved CI care, as indicated by Pisoni et al. [2]. Therefore, we looked for an approach that could specifically explain the differences between good and poor preoperative speech comprehension. For this purpose, the categorised postoperative outcome measure of ‘good’ and ‘poor’ performers was introduced. When this was used, all variables that tested hearing (4FPTA, WRS65(HA), WRS80(HA), WRSmax, NRSmax, SRT with numbers) showed significant association with WRS70(CI). In contrast to the continuous outcome, WRS65(HA) was also found to be significantly correlated with WRS70(CI) in the dichotomous analysis. Nevertheless, the analysis of the continuous WRS70(CI) was pursued as well, since this analysis used the full dataset, which was not possible for the categorised outcome, as the latter analysis ignored the data between the first and third quartiles. Furthermore, comparability with other studies was made possible by using the continuous WRS70(CI), while there is a paucity of literature concerning the approach using a categorised target variable.

Overall, the positive association between preoperative speech comprehension and postoperative outcome observed by other research groups [3,15,21,24,27] was confirmed in this study. The predictive value is limited by the high proportion of patients with 0% word comprehension. However, the graphical analysis showed that the entire range of postoperative speech comprehension is covered (Figure 4). Hoppe et al. addressed this problem by grouping the preoperative data [4]. Consequently, specific observation of individual groups could be performed, but not a continuous statistical analysis for the entire cohort. 4FPTA offered the benefit that only few patients reached a non-measurable range (Figure 4a), with slightly smaller correlation coefficients (ρ = –0.14) compared with WRSmax (ρ = 0.17). Although 4FPTA offers a quick and easy screening method for measuring hearing loss [45,46], and only a few patients reached the non-measurable range, the weak correlation coefficient found in the study of Lazard et al. [24] raises questions about its clinical relevance. The above correlation may not be clinically significant, but it can still be useful when incorporated into a multivariable model to increase the model’s relevance. However, according to Halpin et al. it does not reflect information-carrying capacity [38]. This could explain why 4FPTA is more weakly correlated with WRS70(CI) than WRSmax and WRS80(HA) are.

### 4.2. Patient-Related Factors

When WRS70(CI) was assessed as a continuous variable, a statistically significant relationship was found for (i) meningitis and (ii) congenital hearing loss. In the dichotomous expression of WRS70(CI), this could be confirmed, and a significant relationship was additionally shown for ISSNHL. The percentage of cases with unknown aetiology was lower in our study (32.2%) than that reported by Blamey et al. (53%) [23]. Consequently, results in our study applied to relatively more cases. A reduction in the number of ‘unknown aetiology’ cases to nearly zero would be desirable. Compared with the study cited [23], the proportion of patients with ISSNHL in our study was greater: at 13.2% it corresponded approximately to the North German prevalence of 11% reported by von Gablenz et al. [47]. Without the classification of the outcome into poor/good performers, the influence of ISSNHL would not have been recognised.

Regarding Figure 2, the variables that are significantly correlated with the WRS70(CI) are mostly from the categories that are frequently represented, thus providing the highest statistical power. However, it is possible that the lack of correlation with other influential variables is due to small sample sizes in certain categories, which may lead to undetected correlations [48,49]. To address this issue, the authors of some studies have chosen to merge categories, although excessive merging may result in less diversity [25]. Alternatively, increasing the number of cases or conducting a specific case-control study may be an effective solution [49,50]. In addition, it should be noted that although some influencing factors may affect hearing [51], they do not necessarily have an influence on speech comprehension with a CI.

### 4.3. Prediction and Risk Factors

Pisoni et al. (2018) discussed the lack of preoperative predictors to narrow down the range of expected postoperative outcomes [2]. For this purpose, two different regression models were set up, which should offer the possibility of individual clarification of the postoperative variation.

The binary logistic regression model was used to differentiate the patients below the first quartile from those above the third. The OR referred to the lowest quartile. It resulted in a Nagelkerke’s R^2^ of 0.40 and a Kullback–Leibler divergence-based R^2^ of 0.26. Models with a postoperative variation of 10–26% have been described in the literature [23,24,25,26]. Consequently, and in contrast to the papers cited, the present model explained the postoperative variation at a level above average. However, it must be noted that this binary model uses only half of the data. These were the ears that responded either especially well or especially poorly to the CI. As a result, these ears can easily be differentiated by using a binary logistic regression, because they are at opposite ends of the response spectrum. By using a binary logistic regression model, it can further distinguish between these two groups of ears by examining the impact of various predictor variables on their response status. Such a model would not be suitable for an exact, percentage-based prediction of the result. Nevertheless, it is well suited to identifying influencing or risk factors for a good or poor outcome. The quasibinomial model was additionally employed in order to allow the prediction of WRS70(CI) when all available data for the postoperative target variable (i.e., all patients) were included. The Kullback–Leibler divergence-based R² was 0.23. On the basis of the results presented here, a binary logistic model results in a better postoperative prediction. However each model has its *raison d’être* owing to the different prediction of the target variable and statistical utility.

Regarding the specific predictors, the WRS65(HA) showed a negative correlation with the WRS70(CI) in the quasibinomial model. In terms of the positive correlations between preoperative speech comprehension (WRS80(HA) and WRSmax) and the WRS70(CI), as well as positive correlations with the WRS65(HA) [17,52,53], which were found in other studies, the regression coefficient of the WRS65(HA) should be considered critically. Moreover, the *p* value for WRS65(HA) was higher, and was no longer significant after application of the Bonferroni correction. Besides, the WRS80(HA) and the NRSmax were associated with reduced risk in the multivariate models and thus behaved analogously to the foregoing univariable test. It should be emphasized that in both models WRS80(HA)—and not WRSmax—was found to be the most influential in determining WRS70(CI). Owing to the specific patient population, the coefficients of the present model were primarily valid only for this study. The same applies to the OR found by using the binary logistic regression model. To generalize, further studies outside this cohort should be conducted using the models presented.

In contrast to congenital aetiology and meningitis, ISSNHL was the only aetiology associated with a reduction in the risk of a poor outcome according to the binary logistic regression model. Thus, sensorineural hearing loss represented a beneficial aetiology for CI. While ISSNHL is a common cause of sensorineural hearing loss, it is important to note that other etiologies, such as noise-induced hearing loss and genetic factors, should also be considered. Further, the specific cause of ISSNHL is still unknown, and its diagnosis often relies on the symptoms reported in anamnesis [54,55]. Meningitis is known to be a risk factor in CI outcome [23,24,26] and can affect the brain tissue as well as the cranial nerves, such as the vestibulocochlear nerve [51,56]. The aetiology ‘congenital’ is described in the literature as heterogeneous; a genetic cause can be present in up to 50% of cases [51,57]. This aetiological category includes hypoxia, which can cause severe global brain damage with impaired cognition [58]. In contrast to the findings presented above, early intervention in the first year of life is the preferred and generally accepted intervention [2,43]. The increase in the risk of a poor outcome, as found in this study, was probably due to the association between congenital aetiology and late treatment (after 18 years of age), as well as hypoxic events. It is important to note that we excluded patients under 18 from our study, which suggests that our findings do not account for the effects of late treatment in younger patients. However, our results highlight the importance of early diagnosis and intervention in congenital hearing loss to prevent negative outcomes. Initial age of HA provision influenced WRS70(CI) at older ages in both models. In the quasi-binomial model only, the duration of wearing a HA had an influence: a shorter duration of HA use was associated with better WRS70(CI). A timely provision of an HA and a sufficiently long period of its use are regarded as associated with better hearing ability and later CI provision [39,59]. These variables were not analysed as influencing factors in this study. Older age at HA fitting also means that the hearing ability was previously assessed as not requiring a CI. According to Leung *et al*., onset of deafness at an older age can provide a better hearing situation than life-long deafness [60]. An analogous effect might apply to the duration of HA use. It would be important to clarify which possible confounder influenced the contrary results to literature regarding the initial adjustment and duration of wearing a HA.

The median absolute error for the prediction of WRS70(CI) (see above, “Merging the predictors”) found in a study by Hoppe et al. [17] was 13.5% using WRSmax, and WRS65(HA) and age at CI insertion. Our value for the median absolute error was 13.8%. Some variables found significantly associated with WRS70(CI) were not finally found in the multivariate models used here. Therefore the increase in numbers of categories within a regression model does not always increase the significance of the explanation of postoperative variation. A variable that had a similar range of its reference value (here: WRS70(CI)) as compared with another variable may not offer any additional value in a multivariate analysis. Consequently, increasing the size of a model only makes sense if this leads to an additional increase in knowledge or like in Goudey et al. [25] mentioned by a more complete dataset. 

### 4.4. Limitations of the Study

In some cases, the WRS70(CI) was changed from the originally specified interval of 2 years after implantation to the 1.5-year value, owing to lack of data. This could suggest deviations from the 2-year value based on the increase in speech comprehension over time. Nevertheless, Holden et al. [44] were able to show that 90% of the WRS to be achieved is already reached after 6 months.

Furthermore, the study did not test for possible confounders that could influence the outcome. Lazard et al. (2012) demonstrated the influence of aetiology and patient age on preoperative comprehension [24].

Some factors could not be analysed retrospectively owing to a limited amount of information in the available study data. For example, it would have been desirable to include creeping versus sudden hearing loss, age at onset of hearing loss and cognition testing. For this purpose, it would in the future be of value to prepare preoperative standardised questionnaires to collect relevant information for future studies. This would reduce dependence on the doctor–patient interview as a data source. In addition, the conduct of a prospective study would be helpful in adapting the setting and patient selection to the study objective.

Additionally, although it has known deficiencies, the Freiburg monosyllabic test has a firm place in the postoperative evaluation of the CI [18,20,21,22]. While newer tests of language comprehension, such as the Oldenburg sentence test, have been developed to provide additional insights [61,62], it is important to note that they serve different purposes than the Freiburg monosyllabic test, which was used as our primary evaluation tool. The availability of different tests can offer a more comprehensive understanding of postoperative variability in speech comprehension.

## 5. Conclusions

While general associations of untreated hearing loss with dementia, depression, anxiety and increased mortality [63,64,65] have been described, clarification of the expected individual benefit before CI surgery is relevant. This is of major importance for patients with a preoperative speech comprehension above 0%, because—in contrast to patients without speech comprehension—they may have the choice of continuing to use the hearing aid with residual speech comprehension. In this context, and regarding the identification of risk factors and predictors, the following results were found in the present study:

- Good postoperative comprehension was usually associated with good preoperative comprehension.
- Preoperative WRSmax and WRS80(HA) were better predictors of CI-aided comprehension than was preoperative speech comprehension at 65 dB with HA.
- WRS80(HA) can serve as a useful extension to the usual test level of 65 dB_SPL_ in future preoperative diagnostics.
- A search for aetiological predictors apart from audiological measurement procedures is recommended.
- Our model explained 40% of the postoperative variability under the newly introduced categorised target variable (WRS70(CI) (below the 1st quartile vs. above the 3rd quartile) in the regression model.
- Preoperatively, standardised medical history forms should be used to remedy the lack of data and to reduce the number of unknown aetiologies.
- Future validation of the above models, using independent cohorts, is to be recommended.

The search for variables that can explain and predict interindividual differences in postoperative outcomes is a task for future research [2]. Such investigations could open up perspectives for improving patient counselling and introducing possible alternative measures for patients whose predicted postoperative outcome is relatively poor. In addition to the sometimes uncertain preoperative estimation of the benefit of implanting a CI, a regular update of possible influencing factors could be relevant, as the relevance of individual categories may change over time owing to clinical and scientific progress [23].

## Figures and Tables

**Table 1:** Pre- and postoperative speech comprehension.

	*n*	Median (IQR)	Mean Value	*n* with WRS = 0%
Age at surgery [years]	664	58 (42–71)	56	-
Preop. 4FPTA [dB_HL_]	654	107.0 (93–118)	105.1	-
Preop. WRS65(HA) [%]	635	0 (0–0)	4.2	483
Preop. WRS80(HA) [%]	630	0 (0–20)	12.0	335
Preop. WRSmax [%]	594	0 (0–20)	11.8	334
Preop. NRSmax [%]	605	50.0 (0–70)	42.2	175
Preop. SRT of two-digit numbers [dB_SPL_]	604	115.0 (97.8–130)	111.4	0
Postop. WRS70(CI) [%]	538	75.0 (55–85)	68.0	7

*n* = 664 ears, i.e., the total number of ears in the study. IQR, interquartile range; 4FPTA, four-frequency pure-tone average; WRS65, 80, 70, word recognition score at 65, 80 or70 dB_SPL_ with hearing aid (HA) or cochlear implant (CI); WRSmax, maximum word-recognition score; NRSmax, maximum two-digit number-recognition score; SRT, speech reception threshold.

**Table 2:** Tabular representation of the results of the chi-square test statistics (etiology and categorical EV70CI). MMR, mumps-measles-rubella. Miscellaneous, data that could not be ascribed to a single category. ISSNHL, Idiopathic sudden sensorineural hearing loss.

Etiology Categories	Categorical WRS70(CI)		
	Chi Square Test		
	X²	*p* Value	Cramer-V
Unknown	2.32	0.13	0.08
Ménière's disease	0.78	0.38	0.05
Genetics	4.06	0.04	0.11
General infection	0.35	0.55	0.03
Syndrome complexes	0.22	0.64	0.03
ISSNHL	8.89	0.003	0.17
Meningitis	25.33	<0.0001	0.28
Miscellaneous	0.16	0.69	0.02
Toxicity	0.11	0.74	0.02
Middle ear	5.45	0.02	0.13
Congenital	23.75	<0.0001	0.27
MMR	1.37	0.24	0.07
Trauma	0.11	0.75	0.02

**Table 3:** Comparison of pre- and postoperative speech comprehension in percentage and absolute number of cases. Data for test-retest reliability and for fluctuating performance are reviewed. See text for further explanation.

Comparison between WRS70(CI) and:	WRS70(CI) was Equal or Higher	WRS70(CI) was Lower	WRS70(CI) was Lower after Data Review
WRSmax (Figure 4b)	97.3% (471/484)	2.7% (13/484)	0.6% (3/484)
WRS65(HA) (Figure 4c)	99.2% (512/516)	0.8% (4/516)	0.0% (0/516)
WRS80(HA) (Figure 4d)	97.7% (500/512)	2.3% (12/512)	0.2% (1/512)

**Table 4:** Odds ratio after logistic regression with outcome above the highest and below the lowest quartile of WRS70(CI). OR, odds ratio for negative outcome (i.e., presence within the lowest 25% of WRS70(CI)); 95% CI, 95% confidence interval of the OR; ISSNHL, idiopathic sudden sensorineural hearing loss; HA, hearing aid; NRSmax, maximum two-digit number recognition score.

Influence Variable	OR (95% Confidence Interval)	*p*
(Intercept)	–	<0.001
WRS80(HA)	0.97 (0.94–0.99)	0.005
NRSmax	0.99 (0.98–0.99)	0.038
Meningitis	50.28 (4.71–536.29)	0.001
Congenital & hypoxia	4.68 (1.51–14.51)	0.007
ISSNHL	0.34 (0.11–1.02)	0.041
Age at HA provision		
Below 1st quartile	1 (reference)	
1st–2nd quartile	0.30 (0.13–0.68)	0.004
2nd–3rd quartile	0.24 (0.10–0.57)	0.001
Above 3rd quartile	0.32 (0.13–0.78)	0.012

**Table 5:** Regression for a quasibinomial model with outcome WRS70(CI) in percent. HA; hearing aid.

Influence Variable	Regression Coefficient	Standard Error	*p*
(Intercept)	–2.07	0.30	<0.001
WRS65(HA)	–0.02	0.008	0.014
WRS80(HA)	0.02	0.004	<0.001
Meningitis	–1.30	0.23	<0.001
Congenital & hypoxia	–0.90	0.18	<0.001
Age at HA provision			
Below 1st quartile	Reference		
1st–2nd quartile	0.35	0.14	0.012
2nd–3rd quartile	0.60	0.16	<0.001
Above 3rd quartile	0.69	0.19	<0.001
Duration of HA use			
Below 1st quartile	0.14	0.19	0.45
1st–2nd quartile	0.19	0.16	0.23
2nd–3rd quartile	0.54	0.14	<0.001
Above 3rd quartile	Reference		

## Data Availability

Data available on request due to restrictions eg privacy or ethical. The data presented in this study are available on request from M.H.
